# Vδ2+ and α/Δ T cells show divergent trajectories during human aging

**DOI:** 10.18632/oncotarget.10096

**Published:** 2016-06-15

**Authors:** Crystal Tze Ying Tan, Kilian Wistuba-Hamprecht, Weili Xu, Ma Schwe Zin Nyunt, Anusha Vasudev, Bernett Teck Kwong Lee, Graham Pawelec, Kia Joo Puan, Olaf Rotzschke, Tze Pin Ng, Anis Larbi

**Affiliations:** ^1^ Singapore Immunology Network (SIgN), Agency for Science Technology and Research (A*STAR), Biopolis, Singapore; ^2^ Department of Internal Medicine II, Centre for Medical Research, University Medical Center, Tübingen, Germany; ^3^ Department of Dermatology, University Medical Center, Tübingen, Germany; ^4^ School of Biological Sciences, Nanyang Technological University, Singapore; ^5^ Gerontology Research Programme, Department of Psychological Medicine, National University Health System, Yong Loo Lin School of Medicine, National University of Singapore, Singapore; ^6^ Department of Microbiology, National University of Singapore, Singapore

**Keywords:** unconventional T cells, immunosenescence, phenotype, aging, Gerotarget

## Abstract

Chronological aging and a variety of stressors are driving forces towards immunosenescence. While much attention was paid to the main T cell component, α/β T cells, few studies concentrate on the impact of age on γ/δ T cells' characteristics. The latter are important players of adaptive immunity but also have features associated with innate immunity. Vδ2+ are the main component of γ/δ while Vδ1+ T cells expand upon Cytomegalovirus (CMV) infection and with age. The Vδ2+ T cells are not influenced by persistent infections but do contribute to immunosurveillance against bacterial pathogens. Here, we focus on Vδ2+ T cells and report that their composition and functionality is not altered in older adults. We have performed a side-by-side comparison of α/β and Vδ2 cells by using two robust markers of T cell replicative history and cell differentiation (CD28 and CD27), and cytokine secretion (IFN-γ and TNF-α). Significant differences in Vδ2 versus α/β homeostasis, as well as phenotypic and functional changes emerged. However, the data strongly suggest a sustained functionality of the Vδ2 population with age, independently of the challenge. This suggests differential trajectories towards immunosenescence in α/β and Vδ2+ T cells, most likely explained by their intrinsic functions.

## INTRODUCTION

In the field of aging, many studies have shown a relationship between age, persistent infections and immune profiles [1]. The reduced ability of the elderly to respond to new infections, vaccines or persistent antigenic stress has been termed “immunosenescence”. α/β T cells play important role in mediating immunity to infections whereas the role of γ/δ T cells are less well defined. γ/δ T cells have been implicated in a number of immune responses including immunosurveillance, cytotoxicity, regulation of inflammation and pathogen clearance [2]. γ/δ T cells represent approximately 1-10% of all circulating T cells [3] and they differ from α/β T cells in their unique receptor specificity and different requirements for antigen presenting molecules. In contrast to α/β T cells, antigen recognition by γ/δ T cells is independent of classical major histocompatibility complex (MHC) molecules. γ/δ T cells interact with a broad range of molecules similar to innate immune recognition of pathogen associated molecule patterns [4, 5]. Among the γ/δ T cells, two main subsets are present in peripheral blood that are identified by their delta chains, namely the Vd1 and Vδ2. The latter usually represent the majority (60-80%) of γ/δ T cells whereas Vd1 T cells with only 15 - 20% in the peripheral blood of most healthy adults. Vδ2 T cells are activated by non-peptide phosphoantigens and these include isopentenyl pyrophosphate (IPP) and (*E*)-4-Hydroxy-3-methyl-but-2-enyl pyrophosphate (HMBPP) both serve as metabolic precursors of isoprenoid compounds. Recent studies using genetic approaches have demonstrated that stimulation of Vδ2 T cells by these phosphoantigens is mediated by binding with butyrophilin (BTN) 3A1 [5, 6, 7] Other Vδ2 activators include synthetic drugs aminobisphosphonates that are known for their bone anti-resorptive property. In addition, naturally occurring compounds such as alkylamines that are present in some foods and produce by some bacteria can stimulate Vδ2 T cell expansion.

Unlike Vδ2 T cells, Vd1 T cells are not activated by IPP, HMBPP, aminobisphosphonates nor alkylamines but instead by stress-induced ligands including MIC-A, MIC-B and UL16 binding proteins (ULBPs) [8, 9]. These self-ligands can be upregulated during oxidative stress and after malignant transformation [10]. Since Vd1 T cells express perforin and granzyme, they can exhibit cytotoxicity to epithelial tumors cells and certain leukemia cell lines in a NKG2D-dependent manner [11, 12]. In addition, an increase of Vd1 population has been reported in patients with cytomegalovirus (CMV) infection after transplantations [13].

Chronic and persistent infections caused by CMV, hepatitis B virus (HBV), Epstein-Barr virus (EBV), and human immunodeficiency virus-1 (HIV-1) can result in phenotypic and functional changes in T cell responses leading to T cell exhaustion [14]. T cell exhaustion is characterized by a reduced ability to produce TNF-α, IFN-γ, IL-2, reduced cytolytic activity, and reduced proliferative capacity [15] This phenomenon is well studied in both CD4+ and CD8+ T cells. Whether similar exhaustion can occur in γ/δ T cells need to be determined. Similarly, aging in α/β T cells cell can also result in diminished proliferative capacity (replicative senescence) and a decrease in IL-2 production [16]. We have previously demonstrated that a reduced frequency of /d T cells but not α/β T cells is observed during aging [17]. Here, we extend our study to determine the phenotypic and functional changes of α/β and Vδ2 T cell compartments in the elderly and young individuals.

## RESULTS

### Distribution and phenotype of Vδ2+ and Vδ2-T cells in relation to CMV and aging

**Figure 1:**
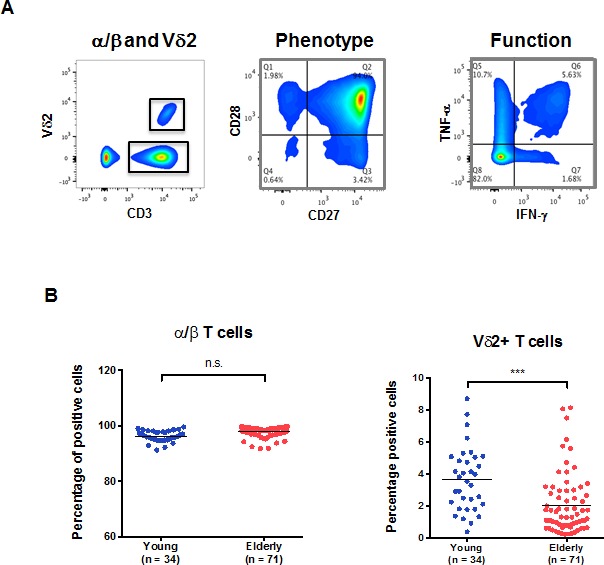
α/β and Vδ2+ T cells in aging PBMCs from young and old individuals were stained and analyzed by flow cytometry. **A.** The gating strategy used to identify the populations of interest in our study is depicted. A typical CD28/CD27 expression profile of T cells (middle panel) and a representative experiment showing the result of T cell stimulation on IFNγ and TNFα intracellular expression (right panel) are shown. **B.** The frequency of total α/β T cells in PBMC from young (blue) and old individuals (red). The frequency of Vδ2+ T cells in young and old individuals within the CD3+ compartment is shown (***, *p* < 0.001).

The identification of the main γ/δ T cells was accomplished using a Vδ2-specific antibody. In Figure 1A, we describe the flow cytometry analysis for the identification of CD3+Vδ2+ versus the rest of the T cell populations. We have designated this latter population α/β T cells throughout the manuscript despite the possible presence of other unconventional T cells (Vd1, MAIT cells), because the CD4+ and CD8+ α/β T cell populations represent >90% in this pool. Standardized methods for exclusion of false positive events were applied and we could clearly distinguish γ/δ T cells expressing the Vδ2 isoform of the TCR. We display the phenotypic characterization of the cells using CD28 and CD27 as well as the functional characterization by staining intracellularly for IFN-γ and TNF-α, respectively. We found that the frequency of total α/β T cells in peripheral blood mononuclear cells (PBMC) was not altered by age (Figure 1B). However, we show a significant reduction of the frequency of V2+ T cells with aging, which is not influenced by CMV seropositivity, as expected [18]. The frequency of Vδ2+ T cells in the young individuals was significantly higher as compared to the elderly individuals. The median frequencies of the young and elderly were 3.68% ±1.99 and 2.03% ±1.86 respectively.

**Figure 2:**
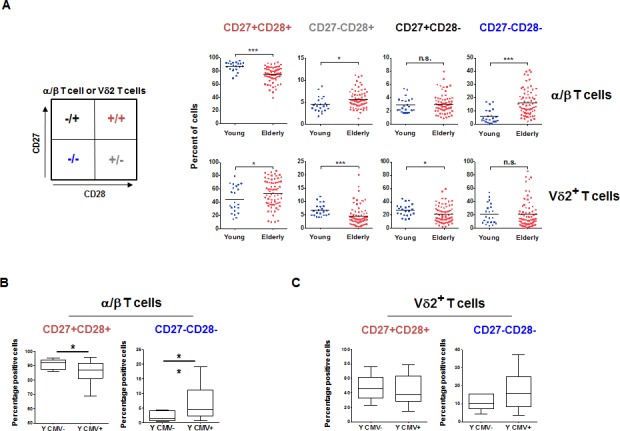
α/β and Vδ2+ T cells subsets aging **A.** The phenotype of PBMC from young and elderly individuals was analyzed by flow cytometry and reported by frequencies of CD28+CD27−, CD28−CD27+, CD28−CD27− and CD28+CD27+ cells in the α/β and Vδ2+ compartments. Significant differences are shown by *p < 0.05, **p < 0.01 and ****p < 0.0001. **B.** The frequency of the less differentiated CD28+CD27+ and most differentiated CD28−CD27− α/β T cells were reported for young individuals based on their CMV serostatus. **C.** A similar analysis was performed for Vδ2+ T cells.

Although the Vδ2+ T cells displayed a significantly different profile, their trajectory with aging is clearly divergent (Figure 2A). The proportion of potentially terminally differentiated α/β T cells (CD28−CD27−) was significantly higher in the elderly compared to the young, a phenomenon not observed for Vδ2+ T cells (Figure 2, right panels). A lower frequency of CD28−CD27+ (*p* < 0.01) and CD28+CD27− (*p* < 0.0001) Vδ2+ T subsets was observed in the elderly. CD28+CD27+ Vδ2+ T cells were over-represented in the elderly as compared to the young (*p* < 0.05). While the majority of α/β T cells expressed CD28 and CD27 in young individuals (mean = 86%, range 69%-96%) this was much less and more variable in the Vδ2+ compartments (mean = 42%, range 16%-79%). As there was no difference in the frequency of Vδ2+ based on CMV seropositivity in young individuals, (3.71% ±2.03 and 3.66% ±2.03) we tested whether there could still be a subset skewing. As expected, there was a higher proportion of the CD28− CD27− late differentiated α/β T cells in CMV positive young donors. However, there was no significant difference for the Vδ2+ T cells (Figure 2B and 2C, respectively).

### Functionality of Vδ2+ and α/β T cells in aging

**Figure 3:**
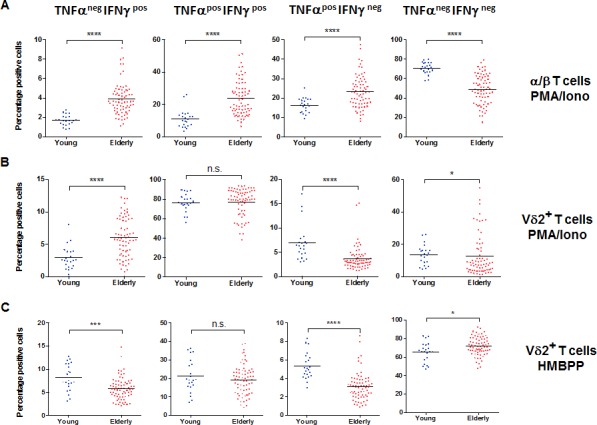

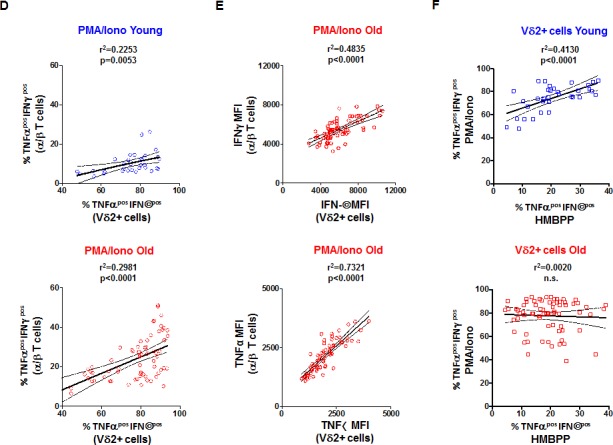
Vδ2+ T cell functional capacity in aging PBMC from young and old individuals were stimulated with 10 ng/ml PMA and 1 mM of ionomycin in the presence of GolgiPlug for 4 hr. **A.** The frequency of TNFα^neg^IFNγ^pos^, TNFα^pos^IFNγ^pos^, TNFα^pos^IFNγ^neg^, and TNFα^neg^IFNγ^neg^ α/β. T cells is shown for young and old individuals. Significant differences are shown by *p < 0.05, ***p < 0.001 and ****p < 0.0001. Similar analysis was performed for samples stimulated to measure functionality following PMA/Ionomycin **B.** or HMBPP **C.** stimulation of Vδ2+ T cells. **D.** The responses were compared between α/β and Vδ2+ T cells and the frequency of poly-functional TNFα^pos^IFNγ^pos^ was calculated in young (top panel) and old individuals (bottom panel). **E.** The mean fluorescence intensities (MFI, a semi-quantitative measure) for TNFα and IFNγ were compared in the TNFα^pos^IFNγ^pos^ of α/β and Vδ2+ T cells from old individuals. **F.** The correlation between TNFα^pos^IFNγ^pos^ frequencies in PMA/Ionomycin versus HMBPP stimulated Vδ2+ T cells in young (top panel) and old donors (bottom panel). R square (r^2^) values and corresponding *p* values are shown in the graphs.

Because α/β T cells do not respond to HMBPP, we tested the overall capacity of Vδ2+ and α/β T cells after mitogenic stimulation (Phorbol 12-myristate 13-acetate (PMA)/Ionomycin). In the case of α/β T cells, we observed a higher overall capacity in the older adults, as shown by their increased ability to produce either TNF-α or IFN-γ, as well as both double positive for TNF-α and IFN-γ (*p* < 0.0001 for each, Figure 3A). We show in Figure 3A and 3B that for the majority of the analyzed individuals, the Vδ2+ T cells are generally more responsive (TNF-α^pos^ IFN-γ^pos^) than α/β T cells. For the same concentration of stimuli, Vδ2+ T cells show a powerful response, with >75% of the cells able to produce both TNF-α and IFN-γ, independently of age (*p* > 0.05, Figure 3B second panel). For single cytokine production, we observed that Vδ2+ T cells from older individuals have a higher ability to produce IFN-γ only (*p* < 0.0001, Figure 3B first panel) but have lower proportions of cells able to produce TNF-α only (*p* < 0.0001, Figure 3B third panel). Again, these two populations represent a minority of the responding cells (≈ 5%). We also used HMBPP as a stimulatory agent for the activation of Vδ2+ T cells. There was a slightly higher frequency of non-responding Vδ2+ T cells in the elderly (*p* < 0.05, Figure 3C, right panel). We identified this as not being caused by a reduced ability to produce both IFN-γ and TNF-α but by a reduced frequency of single TNF-α^pos^ and single IFN-γ^pos^ producers (*p* < 0.001, *p* < 0.0001 respectively, Figure 3C). Of note, these single functional cells represent a very small fraction of all responding cells. Thus, with aging, Vδ2+ T cells do not show loss of the co-stimulatory molecules, and tend to maintain a high level of functional capacity. We display in Figure 3D data showing that despite a possible divergent trajectory between Vδ2+ and α/β T cells with increasing age, there is still some correlation between the overall frequency of TNF-α^pos^ IFN-γ^pos^ cells in Vδ2+ and α/β T cells, independently of age. The amount of cytokines produced per cell, measured by mean fluorescence intensity (MFI) is strongly correlated in the two populations, especially for TNF-α (Figure 3E, r^2^ = 0.7321, *p* < 0.0001) in response to PMA/Ionomycin. The same applied to TNF-α and IFN-γ in young individuals (r^2^ = 0.6806 *p* < 0.0001 and r^2^ = 0.6482 *p* < 0.0001, respectively). We could not compare the response to HMBPP, as α/β T cells do not respond to this stimulus. Finally we tried to understand whether the mitogen-induced overall capacity of Vδ2+ T cells (with PMA/Ionomycin) could be related to the TCR-dependent specific response (with HMBPP). We observed (Figure 3F) that in young individuals there is a significant correlation between the two (r^2^ = 0.4130, *p* < 0.0001) but this is not observed in the old individuals (r^2^ = 0.0020, p>0.05). This further suggests that there is a dichotomy between phenotype and functionality switch with aging in α/β versus Vδ2+ T cells.

### Kinetic and intensity of Vδ2+ responses

**Figure 4:**
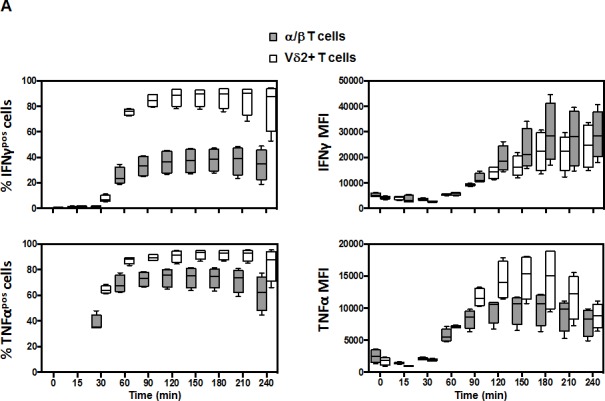

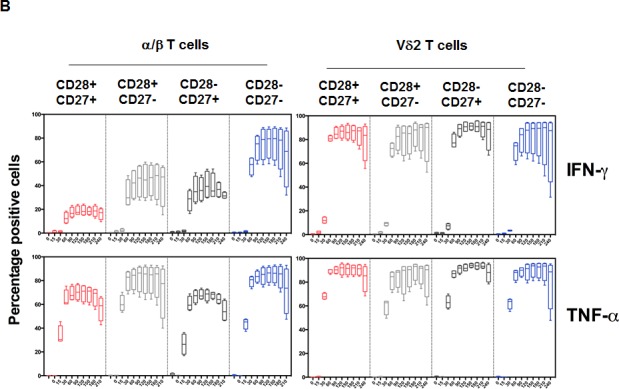
Kinetic response of Vδ2 and associated subsets PBMC from healthy young donors (*n* = 4) were stimulated with PMA (10 ng/mL) and Ionomycin (1 mM) in the presence of GolgiPlug for the indicated time-points. The levels of TNFα and IFNγ were determined by intracellular cytokine staining after 15, 30, 60, 90, 120, 150, 180, 210 and 240 minutes stimulation. **A.** Significant differences in the magnitude of IFNγproduction between Vδ2+ and α/β T cells were observed for all times points after 30 min (top panels, *p* < 0.0001). Similar profiles and differences were observed for the TNFα response (lower panels). The MFI for IFNγ was not significantly different between Vδ2+ and α/β cytokine-producing cells while it was different for the TNFα MFI for the 60-210 minutes time-points. **B.** The same analysis was performed using CD28 and CD27 markers to discriminate the subsets. The graphs show the frequency of responding cells in each subset for the corresponding cytokine. There were no significant difference in the magnitude and kinetic of response in the difference CD28/CD27 subsets of Vδ2+ T cells contrary to α/β T cells.

Based on our findings, we examined the differential cytokine response between Vδ2+ and α/βT cells could be caused by a difference in the kinetic. We performed PBMC stimulation with PMA/Ionomycin and measured the expression of TNF-α and IFN-γ every 30 min (Figure 4A). We observed that the frequency of total TNF-α^pos^ and IFN-γ^pos^ cells was increased very rapidly in both Vδ2+ and α/β T cell populations. The responses plateaued after 90 minutes of stimulation for both populations. This suggested that the difference observed between Vδ2+ and α/β T cells was not due to a difference in the kinetics. TNF-α and IFN-γ responses showed very similar profiles in Vδ2+ T cells. However, α/β T cells were more capable of producing TNF-α than IFN-γ (Figure 4A, left panels). We confirm here (Figure 4, right panels) the suggested correlations of functionality at the single cell level (measured by mean fluorescence intensity) between Vδ2+ and α/β T cells. Despite the large difference in the number of cells producing IFN-γ, there was little difference regarding IFN-γ secretion at the single cell level based on MFI between α/β and Vδ2+ T cell populations (Figure 4A). This semi-quantitative analysis also shows that there is a steady production of both IFN-γ and TNF-α that plateaued after 180 min. Overall, the capacity of both populations is fairly similar; only Vδ2+ T cells produced more TNF-α between 60 min and 210 min (range: +15% to +40%). In light of these results, we tested the hypothesis that there was no subset-specific production of IFN-γ and TNF-α in Vδ2+ T cells because the response was so high, contrary to α/β T cells. To assess this, we gated the populations based on CD28/CD27 expression. In Figure 4B we demonstrate that both IFN-γ- and TNFα-producing Vδ2 T cells showed a very similar profile for all CD28/CD27 subsets (right graphs). However, there is a distinct profile with α/β T cells. Late differentiated (CD28−/CD27−) α/β T cells showed a higher capacity to produce IFN-γ (left graphs) compared to early memory cells (CD28+/CD27+). A similar profile was observed for TNF-α expression, although less dramatically different (Figure 4B). Thus, differential responses between Vδ2+ and α/β T cells are unlikely to be due to modulation of the responsive subset over time.

### Vδ2 T cell responses in old age

**Figure 5:**
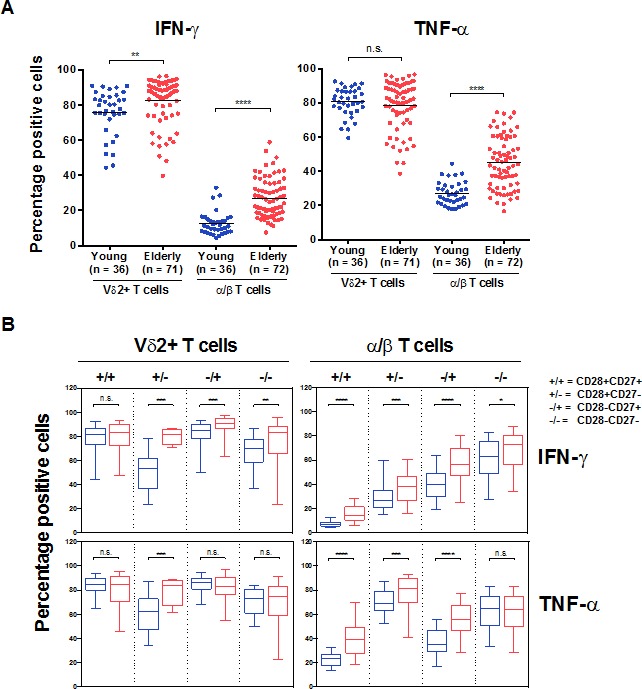

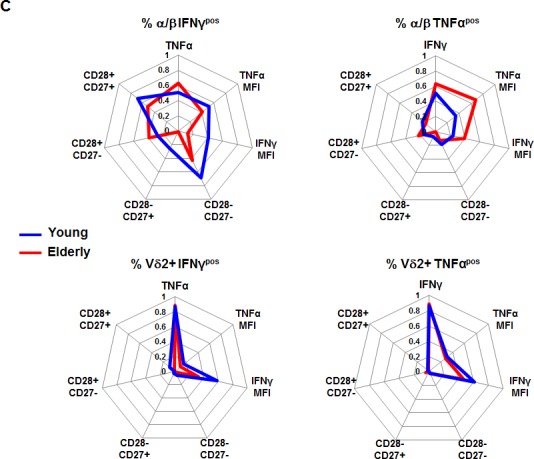
Vδ2+ versus α/β T cells functional capacity in aging PBMCs from young and old individuals were stimulated with 10 ng/mL PMA and 1 mM of ionomycin in the presence of GolgiPlug for 4 hours. **A.** Total and **B.** CD28/CD27 subsets of T cells are shown. Significant differences in the response between young and old individuals are shown by *p < 0.05, **p < 0.01, ***p < 0.001 and ****p < 0.0001. **C.** Radar display of Pearson's correlations (r^2^ values) between the frequency of responding Vδ2+ and α/β T cells (TNFα^pos^ or IFNγ^pos^) and their respective features in young and elderly individuals.

Knowing that responses in Vδ2+ T cells are independent of their CD28/CD27 profile and that kinetic studies show no time effect, we further show here that in elderly individuals a very high levels of cytokine production capacity is maintained (Figure 5A). The higher frequency of cytokine-producing α/β T cells in the elderly mainly reflects the higher frequency of cells lacking one or two co-receptors (CD27^−^ and/or CD28^−^ representing Effector Memory or Terminal Effector cells). We distinguished the phenotype of IFN-γ^+^ cells and demonstrate that every subset of α/β T cells, from the early (CD28+CD27+) to late stage differentiated (CD28−CD27−), showed steadily increasing ability to produce IFN-γ (Figure 5B). The profile of IFN-γ^+^ Vδ2+ T cells was clearly less dependent on CD28/CD27 expression. A similar phenomenon has been identified for TNF-α (Figure 5B), while α/β cells lacking CD27 expression are as potent as Vδ2+ cells regarding TNFα production.

Finally, we aimed to identify the relationship between responses in V2 and other intrinsic markers. The Pearson r^2^ values between the frequency of functional cells measured by IFN-γ and TNF-α response, phenotypic and functional markers are displayed in a net diagram (Figure 5C), for young and elderly individuals. The main observation from this diagram is that α/β T cell relationships of IFN-γ^+^ differ from TNF-α^+^ and that aging differentially impacts on this (upper diagrams). For instance, the overall IFN-γ response can be predicted by the phenotype of the α/β T cells (especially frequency of CD28+CD27+ and CD28−CD27− T cells). In contrast, Vδ2+ T cells show no differences between IFNg and TNFα and no effect of aging. Finally, we tested the hypothesis that Vδ2+ T cell responsiveness could be predicted by α/β T cell-related markers (Table 1). We aimed to test this to identify whether previously published work could be used to investigate potential changes in Vδ2+ T cells in other cohorts. However, neither CD28 nor CD27 expression in α/β T cells and Vδ2+ T cells was able to directly predict the functional capacity of the other population and vice-versa (Table 1). Nevertheless, the higher quartile response in Vδ2+ T cells could be predicted using the frequency of TNF-α^+^ and IFN-γ^+^ T cells independently of age.

**Table 1:** Relationship between Vδ2+ and α/β responses

α/β	Vδ2+	Adjusted *p* value
OLD % TNFα+	OLD TNFα quartile	1.79E-07
YOUNG % TNFα+	YOUNG TNF quartile	8.46E-03
OLD TNFα MFI	OLD TNFα quartile	3.59E-04
YOUNG TNFα MFI	YOUNG TNF quartile	2.53E-03
OLD % TNFα+	OLD IFNγ quartile	2.65E-05
YOUNG % TNFα+	YOUNG IFNγ quartile	2.61E-01
OLD TNFα MFI	OLD IFNγ quartile	2.68E-03
YOUNG TNFα MFI	YOUNG IFN quartile	2.45E-02
OLD % IFNγ+	OLD IFNγ quartile	2.53E-03
YOUNG % IFNγ+	YOUNG IFNγ quartile	3.25E-02
OLD % IFNγ MFI	OLD IFNγ quartile	2.93E-05
YOUNG % IFNγ MFI	YOUNG IFNγ quartile	2.53E-03
OLD % IFNγ+	OLD TNFα quartile	1.30E-03
YOUNG % IFNγ+	YOUNG TNFα quartile	3.71E-03
OLD % IFNγ MFI	OLD TNFα quartile	4.40E-06
YOUNG % IFNγ MFI	YOUNG TNFα quartile	2.53E-03

Responses of α/β and Vδ2+ T cells to stimulation were compared. Higher quartile response in Vδ2+ T cells could be predicted using the frequency of TNFα^+^ and IFNγ^+^ T cells independently of age.

## DISCUSSION

The immune system possesses a myriad of sensors that allow its activation and adaptation to a wide range of stressors. Challenges encountered during the lifespan directly influence the differentiation of immune cells, especially T cells, and particularly when infections are persistent, such as CMV and HIV induce what is sometimes considered to be accelerated immunological aging [19]. Here we compare the quantitative and qualitative differences of α/β and Vδ2+ T cells between the young and elderly cohorts. Whilst there is no difference in the frequency of α/β T cells between young and elderly, there is a significant difference in the Vδ2+ T cells. In the elderly individuals, there is a lower frequency of Vδ2+ T cell in the elderly compared to the young. This decline in Vδ2 frequency is caused by a reduction in Vδ2 absolute cell count and consistent with previous studies [20, 21]. In a recent study it was reported that the reduction in Vδ2 frequency was significant only in old CMV+ individuals and not in the young population, as we do report here. The expansion of Vd1+ T cells observed in this context is interesting but should be interpreted with care as the frequency of total γ/δ T cells is reduced with age, and the Vd1/Vδ2 ratio and number provide different information [22]. Moreover, it has previously been demonstrated that Vδ2 T cell population is likely to undergo activation-induced cell death *in vitro* compared to Vd1 T cells following mitogen stimulation [21]. In any case, there is an age-associated specific loss of Vδ2 T cells in the elderly.

Vδ2 T cells comprise of a heterogeneous population in the circulation that can be divided into 4 subsets based on the surface expression of CD27 and CD28 co-activator for T cell activation. An altered distribution of the γ/δ T cells has been described in certain infections and solid malignancies [23–26]. Our result shows that there is broad variation in the CD27 and CD28 double positive Vδ2 T cells in both the young and elderly cohorts. There is a reduced frequency of CD27+CD28+ Vδ2 T cells in the elderly compared to the young. But there is no difference in the CD27 CD28 double negative Vδ2 T cells in both cohorts. In contrast to Vδ2 T cells, CD27+CD28+ double positive cells are reduced in α/β T cells in the elderly and this is accompanied by a preferential increase of CD27−CD28+ and CD27−CD28− double negative α/β T cell subsets. The increase of CD27−CD28− α/β T cells is attributed to the CMV sero-positive status. CMV positivity has little impact on the skewing of the CD27− CD28− Vδ2 T cells. Our result is consistent with previous finding showing that CMV has stronger effect on Vd1 or Vδ2- T cells [27].

CD4 and CD8 α/β T cells with simultaneous production of multiple cytokines and degranulation are of interest because of their association with anti-viral responses. Studies on HIV patients have shown that CD8 T cells with the capacity to secrete multiple cytokines are associated with improved patient outcome after anti-retroviral therapy [28]. There is little information on the “polyfunctional” Vδ2 T cells. In this study, we measured the single cytokine producers versus the double cytokine producers in α/β T cells and Vδ2 T cells in the young and elderly cohorts. Stimulation of α/β T cells using the polyclonal activators, PMA and ionomycin, showed an increased of TNF-α and IFN-γ double positive α/β T cells in the elderly compared to the young. Previous studies by Van Epps et al. have also demonstrated both CD4+ and CD8+ T cells in elderly displayed higher proportion of polyfunctional phenotype compared to the young [29]. In contrast to the α/β T cells, no difference is observed in both cohorts in the Vδ2 T cells after stimulation. Interestingly, in the young individuals the TNF-α and IFN-γ double positive Vδ2 T cells induced by PMA/ionomycin correlates with double positive induced by HMBPP. This “polyfunctional” correlation disappears in the elderly individuals. PMA and calcium ionomycin stimulate T cells through direct activation of protein kinase C and stimulation of calcium release from endoplasmic reticulum store, thereby by-passing the TCR. HMBPP stimulation of Vδ2 T cells depends on its interaction with Vg2Vδ2 TCR and butyrophilin 3A1 on the antigen presenting cells. We postulate that it is likely that aging could alter binding affinity of either of the V2Vδ2 TCR and/or butyrophiln 3A1 resulting in an impaired Vδ2 T cell response.

Our study also highlights that the investigation of immunosenescence should be broadened to include other T cell populations in order to better understand its clinical importance and biological significance. Future studies aiming at modulating Vδ2+ frequency and functionality may be of clinical value. Recently, it was shown that azathioprine treatment in Crohn's disease patients selectively eliminated Vδ2+ T cells [30], which suggests the possibility of its utilization in settings where dysregulation of V2+ cell frequency/activity is associated with clinical outcomes such as in systemic

lupus erythematosus [31]. Current studies in our laboratory focus on Vd1+ T cells in order to defined whether the reported differences with the α/β T cells also apply. As Vδ2+ T cells show a dichotomy for their differentiation we suggest this population to use differential signaling for this process or that homeostasis of the population is regulated very differently than the classical T cell populations. This could explain the relatively “younger” profile of Vδ2+ T cells or suggest that the signals leading to differentiation and/or senescence are integrated differently. Early studies did show the dramatic loss of phenotypically “naïve” Vδ2+ T cells in the first years of life [32] but its relationship with later outcomes has not been investigated. Studies have indeed shown that during senescence T cells are able to utilize signaling pathways (via TAB1 and p38) that are absent in non-senescent T cells [33]. The possibility that the Vδ2+ T cell population is protected from senescence by an opposite phenomenon should not be excluded. Other studies suggest Vδ2+ T cells to retain capacity for TNFα secretion and we show in the present study that TNFα levels do not vary between CD28/CD27 subpopulations of HMB-PP-activated Vδ2+ T cells (Figure 4B). Li et al. reported that the presence of TNFα (and TNFRII) is a cornerstone for Vδ2+ T cell activation in a paracrine and autocrine manner as well [34]. Together, we can hypothesize that the TNF/TNFR pathway may reduce susceptibility to senescence in T cells, most probably by signaling crosstalk. Studying these aspects in younger cohorts than we had may be informative for possible ways to sustain T cell responsiveness in aging and other conditions mimicking the aging process.

## MATERIAL AND METHODS

### Donors and blood collection

Participants of the study (*n* = 72, 55-85 years old, mean age = 66.1 years, Ethnicity: 14% Indians, 8% Malays, 78% Chinese. Gender: 40% Male, 60% Female) were enrolled in the Singapore Longitudinal Aging Study 2 (SLAS-2). Details the SLAS study design, population sampling and measurements were previously published [34]. In brief, all residents were identified from the door-to-door census and eligible persons without severe physical or mental disability were invited to participate in the study. Individuals with a history of hospitalization in the past 6 months and high C-Reactive Protein (CRP) levels (>3mg/L) were excluded. The young volunteers (*n* = 36, 18-23 years old, mean age = 20.8 years. Ethnicity: 100% Chinese. Gender: 50% Male, 50% Female) were enrolled from the National University of Singapore. The research was approved by the National University of Singapore Institutional Review Board (IRB 10-445), and informed consent was obtained from all participants. Fasting blood was collected and processing started within 2 hours.

### Cell preparation

Blood was collected in Cell Processing Tubes (CPT vacutainers; Becton Dickinson USA) and processed according to the manufacturer's recommendations. The CPT tubes were centrifuged and plasma was collected and aliquoted for storage at −80°C. The Peripheral Blood Mononuclear Cell (PBMC) layer was collected and washed twice with PBS containing 5% FCS and cryopreserved (5 x10^6^ cells/tube) in 10% DMSO with 90% FBS and stored in liquid nitrogen until ready for phenotypic characterization and *in vitro* stimulation. PBMC were rapidly thawed at 37°C, diluted in RPMI containing 10% FCS and subsequently washed three times with PBS containing 5% FCS. Recovery was higher than 75% with viability of >95%.

### PBMC stimulation

PBMC were suspended in stimulation medium (RPMI1640 supplemented with 10% FBS, 2 mM L-glutamine, 1 mM sodium pyruvate, 10 mM HEPES) to assess the cytokine production by Vδ2+ and α/β T cells simultaneously. PBMC were stimulated with 0.316 mM of (*E*)-4-Hydroxy-3-methyl-but-2-enyl pyrophosphate (HMBPP; Echelon Biosciences Incorporated, USA) or 10ng/mL phorbol 12-myristate 13-acetate (PMA; Sigma-Aldrich, USA) together with 1 mM ionomycin (Sigma-Aldrich, USA) in 1.5 mL eppendorf tubes in the presence of 1:1000 GolgiPlug (BD Biosciences, Pharmingen, USA). Stimulation was performed at 37°C for 4 hours unless stated otherwise.

### Flow cytometry

PBMC were stained with LIVE/DEAD Aqua Fixable Stain Kit (L/D Aqua; Molecular Probes, Invitrogen, USA) together with monoclonal antibodies specific for CD3 Pacific Blue (UCHT1), Vδ2 TCR PerCP (B6), CD27 APC (O323) and CD28 PE-Cy7 (CD28.2) for 20 minutes in the dark at 4°C in PBS containing 5% FCS, 2 mM EDTA, and 0.1% sodium azide (FACS buffer). When needed, cells were fixed and permeabilized for 20 minutes at 4°C with BD CytoFix/CytoPerm Fixation and Permeabilization Solution (BD Biosciences, Pharmingen, USA). The cells were washed twice with 1X Perm/Wash Buffer (BD Biosciences, Pharmingen, USA). The cells were then stained in 1X Perm/Wash for anti-human IFNg (4S.B3) and anti-human TNFα (MAb11). Cells were washed twice and resuspended in 200 ml FACS buffer. Samples were acquired in a BD LSRII Fortessa flow cytometer using automatic compensations.

### Serology

Plasma from the participants were thawed and analyzed for the presence of antibodies (IgG) against cytomegalovirus (CMV). This was tested using a semiquantitative ELISA kits (Omega Diagnostics) according to the manufacturer's instructions. Results showed seropositivity for CMV for all elderly individuals while the latter was observed for 24 out of 36 young participants.

### Data analysis

For analysis of flow cytometric data FACSDiva (BD Biosciences) and FlowJo V10.06 (TreeStar, Portland, USA) were used. Statistical analysis was performed using Prism 6 (Graph Pad Software, Inc. La Jolla, USA). For comparisons between two independent groups the Mann-Whitney U Test was used. *P*-values < 0.05 were considered significant.
